# Long-Term Treatment of Highly Saline Brine in a Direct Contact Membrane Distillation (DCMD) Pilot Unit Using Polyethylene Membranes

**DOI:** 10.3390/membranes12040424

**Published:** 2022-04-14

**Authors:** Haneen Abdelrazeq, Majeda Khraisheh, Mohammad K. Hassan

**Affiliations:** 1 Department of Chemical Engineering, College of Engineering, Qatar University, Doha P.O. Box 2713, Qatar; ha082881@student.qu.edu.qa; 2 Center for Advanced Materials, Qatar University, Doha P.O. Box 2713, Qatar; mohamed.hassan@qu.edu.qa

**Keywords:** polyethylene membrane, pure water flux, membrane fouling, specific energy consumption, membrane distillation pilot study

## Abstract

Membrane distillation (MD) is an attractive separation process for wastewater treatment and desalination. There are continuing challenges in implementing MD technologies at a large industrial scale. This work attempts to investigate the desalination performance of a pilot-scale direct contact membrane distillation (DCMD) system using synthetic thermal brine mimicking industrial wastewater in the Gulf Cooperation Council (GCC). A commercial polyethylene membrane was used in all tests in the DCMD pilot unit. Long-term performance exhibited up to 95.6% salt rejection rates using highly saline feed (75,500 ppm) and 98% using moderate saline feed (25,200 ppm). The results include the characterization of the membrane surface evolution during the tests, the fouling determination, and the assessment of the energy consumption. The fouling effect of the polyethylene membrane was studied using Humic acid (HA) as the feed for the whole DCMD pilot unit. An optimum specific thermal energy consumption (STEC) reduction of 10% was achieved with a high flux recovery ratio of 95% after 100 h of DCMD pilot operation. At fixed operating conditions for feed inlet temperature of 70 °C, a distillate inlet temperature of 20 °C, with flowrates of 70 l/h for both streams, the correlations were as high as 0.919 between the pure water flux and water contact angle, and 0.963 between the pure water flux and salt rejection, respectively. The current pilot unit study provides better insight into existing thermal desalination plants with an emphasis on specific energy consumption (SEC). The results of this study may pave the way for the commercialization of such filtration technology at a larger scale in global communities.

## 1. Introduction

In recent years, desalination has become a necessary part of the global resolutions that tackle the issue of water scarcity [1]. Studies have shown that there is a growing demand for the purification of high-level concentrations of wastewater (saline brine), especially that which is generated from existing desalination plants and to reduce its harmful environmental consequences to the minimum [2]. Brine is usually disposed of as a waste product without proper pre-treatment protocols. However, taking into consideration the massive amounts of wastewater generated from the oil and gas industries, the focus on mature and fully developed innovative membrane technologies is being fully considered in terms of energy costs and environmental constraints [3].

Nowadays, many technological advancements in the field of water desalination are majorly focusing on treating seawater or brackish groundwater, while brine with higher salinity (>35,000 mg/L) gained a little amount of consideration and was rarely reported in the literature. Reverse osmosis (RO) is one of the economically widely used wastewater treatment technologies for the purpose of desalinating ultra-high salinity brine (<45,000 mg/L) [2]. Nevertheless, it is not the optimal technology for treating heavily contaminated produced water. Membrane Distillation (MD) technologies, either used alone or in combination with other innovative separation processes, are better alternative options that are designed for complete salt removal. This is because: (i) membrane distillation desalinates thermal brine and generates a permeate that is suitable for immediate reuse [4], and (ii) RO technologies may mostly be used in cases where the salinity of brine is minimized to levels close to that of seawater [5].

Membrane distillation is a temperature-dependent process that functions based on vapor-liquid equilibrium (VLE) and needs a heat source to be supplied to attain the requisite latent heat of vaporization of the feed solution [6]. It is simply a single hybrid process unit consisting of thermal evaporation and membrane separation [7]. Among all MD configurations, characterized by the mode of vapor recovery on the permeate side, the simplest and easiest to operate is Direct Contact Membrane Distillation (DCMD). It is the most studied MD configuration and can be carried out in any desired membrane configuration, such as flat sheet, spiral wound, capillaries, or even hollow fiber [8].

However, the major drawback in membrane-based processes is the buildup of undesirable biomass and calcium residues on the membrane surface from the feed side, which is known as fouling and scaling, respectively. These phenomena hinder the membrane’s hydrophobicity, porosity, as well as pore size acceptable for MD processes [9]. The accumulated foulants may either be natural organic substances (organic compounds) or carbonate and chloride salts (inorganic compounds) [10]. Generally, organic and inorganic fouling are considered major challenges in large-scale membrane distillation processes that must be properly addressed to avoid minimal salt removal [11], increased energy consumption [12], and process shutdown [13]. When using industrial brine as the feed in the water treatment process, microfiltration (MF) and ultrafiltration (UF) techniques are mainly used as pre-treatment methods to reduce the fouling potential of non-dissolved contaminants.

Horseman et al. [14] indicated that gypsum scaling could be prevented by combining the use of superhydrophobic membranes and periodic gas purging in the MD system. Results have not shown any significant flux decline with high effectiveness in eliminating salt crystals from the membrane surface. With changing operating process parameters, membrane scaling can be delayed but cannot be completely avoided. Previous studies have shown that the purging efficiency during the MD scaling mitigation process is highly dependable upon the initial concentration of the brine. Recent MD studies have demonstrated that Humic acid (HA) aggregates play a major role in the fouling process [15,16,17,18,19]. Hence, it was important to study the HA fouling effect on PE in our pilot DCMD system using synthesized thermal brine that mimics industrial wastewater in the GCC region. 

From an industrial viewpoint, The desalination and treatment of high-salinity brines are inherently energy-intensive [20]. Particularly in MD processes, due to latent heat needed for the evaporation of the feed, the energy requirement significantly rises. The criteria behind evaluating the energy performance of an MD system are divided into two main parts: (i) standard measures directly related to the fundamentals of the system, and (ii) developed measures based on the specificity of the employed system [7]. The evaluation of the Specific Energy Consumption (SEC) is a common parameter used to evaluate the energy efficiency of MD systems [21,22]. Although the different types of MD systems are very promising in terms of energy efficiency effectiveness, most of them have not exceeded 10 years of continuous application. Hence, more experimental and theoretical are needed to fully assess the overall MD operations [23].

There have been recent investigations on the operating process parameters in membrane distillation for the treatment of highly saline feeds [24]. However, scaled-up treatment processes in DCMD using industrial wastewater as thermal brines with a focus on the system’s energy consumption and membrane’s fouling behaviors are rarely found in the literature [6]. It is important to provide sustainable solutions that can only be obtained through research and technology development with improved MD systems. This work focuses on optimizing the desalination performance using a pilot-scale DCMD module by utilizing a synthesized feed solution mimicking industrial wastewater. The DCMD performance greatly assists in incorporating the low-cost desalination of wastewater in industrial desalination plants using abundant waste heat from other industrial processes. This cannot be achieved through a conventional bench-scale DCMD setup. Hence, a pilot-scale study using direct contact membrane distillation is implemented with emphasis on the effect of varying brine concentrations, on its energetic performance and with a closer outlook on the effect of membrane fouling over long periods of time. This work involves the operation of an innovative technological pilot unit that can be scaled up in the industry.

## 2. Materials and Methods

### 2.1. Polyethylene Membranes

Large-sized polyethylene membranes with an effective area of 0.01 m^2^ (dimensions of effective area: 175 × 125 mm) were used without further modification from Aquastill, NL. The pore size range of the PE membranes was 300–700 nm with a mean thickness of 15.5 ± 1.53 μm.

### 2.2. Preparation of Feed

An amount of 20 L synthetic brine was prepared as the feed with compositions similar to that in industrial thermal desalination plants in Qatar [25]. The employed feed solutions used in this study consisted of a chemical mixture of calcium chloride dehydrate (purity 99.9%, CAS: 7791-18-6, Manufacturer: Sigma-Aldrich, St. Louis, MI, USA), potassium chloride LR (Code:0001276, Breckland Scientific Supplies, Stafford, UK), magnesium chloride (purity > 98%, CAS: 7786-30-3, Sigma-Aldrich, St. Louis, MI, USA), sodium chloride (purity 99%, CAS: 7647-14-5, Sigma-Aldrich), magnesium sulfate 7-hydrate (purity 99%, CAS: 10034-99-8, Surechem products Ltd.), potassium bromide analytical reagent grade (CAS: 7758-02-3), strontium chloride (CAS: 10476-85-4, Sigma-Aldrich), and boric acid (purity > 99.5%, CAS: 10043-35-3, Sigma-Aldrich). All chemicals were used as received without additional purification [26]. The concentration of each chemical component is listed in Table 1.

### 2.3. Operation of the Direct Contact Membrane Distillation (DCMD) Module

**Figure 1:**
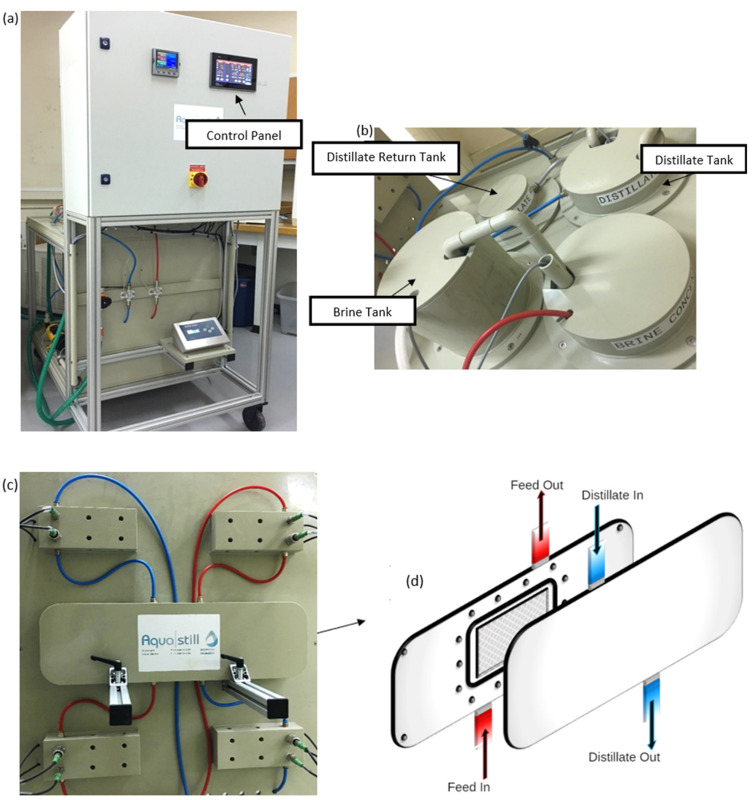
(**a**) Front side of the DCMD pilot unit at Qatar University labs, (**b**) the tanks on the backside of the pilot unit (**c**) Membrane channels from the back side of the pilot unit, and (**d**) 3D illustration of DCMD membrane holder showing counter current flows in the DCMD pilot unit.

A DCMD pilot unit was used for measuring the performance of the polyethylene membranes using synthetic thermal brine. The tanks are made from polypropylene, whereas the pumps are made by Pan World NH-100-PX. Due to the chemical resistance of the build-up materials, this pilot unit is suitable for testing our high-salinity brine with TDS values reaching up to 100 mS/cm. Furthermore, this large unit consists of four temperature sensors, two pressure sensors, and two flow meters. The process parameters are manually entered by tapping on the required setpoint using the electronic control panel located on the front side of the unit, as shown in Figure 1. As for the recording of information, a built-in flash drive inside the pilot unit automatically saves the data each time the pump returns the distillate back to the brine. Electrical heating of 3KW is available inside the feed tank. The heating is automatically controlled and switches off if it exceeds the maximum allowable temperature. An external chiller is directly connected to the distillate tank.

**Figure 2:**
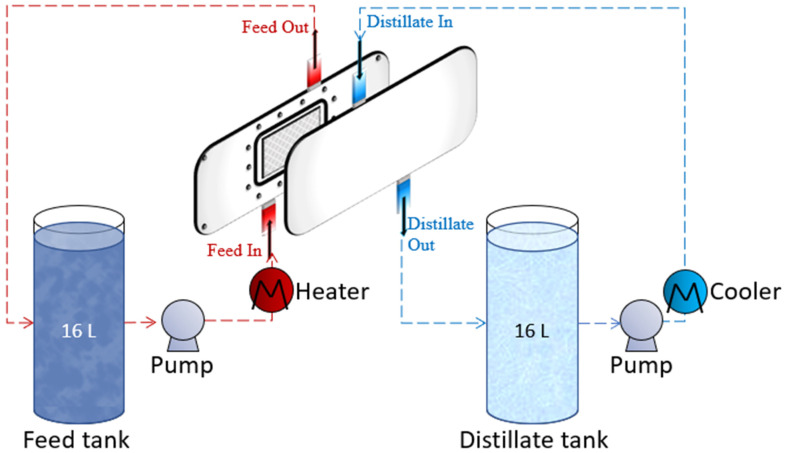
Schematic diagram for DCMD pilot unit.

All experiments were performed using the normal operation mode for long periods of time (reaching up to 100 h). This DCMD pilot unit was used for operational DCMD testing hours of 20, 40, 60, 80, and 100. In the DCMD pilot unit, three chambers were utilized: the brine, distillate, and distillate return tank. The brine tank was filled with 16 L of feed, and the distillate tank was filled with 16 L of distilled water to ensure a temperature gradient across the membrane channels. The distillate produced was collected in the distillate return tank until it reached the maximum level, where the distillate return pump started pouring back the excess amounts of produced distillate back into the brine tank (Figure 2). This DCMD process was assumed to be (i) steady-state, (ii) no heat exchanged with the surrounding (isolated system), and (iii) counter-current flow direction. 

### 2.4. Fouling Analysis for DCMD Pilot Unit

In this work, a Humic Acid sodium salt with technical grade (CAS: 68131-04-4) was purchased from Sigma-Aldrich. A stock solution was prepared, as the foulant, by dissolving an amount of HA in deionized water. A standard concentration of (50 ppm) of Humic Acid was prepared by dissolving HA in DI. The working solutions of required concentration were prepared by sequential dilution of the standard solution. The control foulant concentrations were measured using a UV-visible spectrophotometer at λ_max_ = 600 nm at initial concentrations of 15–45 mg/L. Consequently, the fouling performance of the polyethylene membranes was tested in the pilot-scale DCMD system. The HA rejection was calculated based on the HA concentration obtained by the UV spectrophotometer. The initial HA concentration was measured by taking a sample from the feed before starting the DCMD experiment (the control), and the final HA concentration that is collected from the permeate is the measured value. The rejection values were calculated for different HA concentrations using the difference between the control and measured concentrations divided by the control adsorption. 

### 2.5. MD Performance Evaluation

#### 2.5.1. Salt Rejection and Flux Calculations 

The salt rejection rate (SR) was estimated using the equation [27]:

(1) $$R=\left(1-\frac{C_{p}}{C_{f}}\right)\times 100\%$$

where C_f_ the salt concentration for the feed solution and C_p_ the salt concentration in the permeate solution.

The pure water flux was determined using the permeate volume (V in L), the effective membrane area (A in m^2^), the operation time (t in hours), and the operation pressure (P in MPa) by applying the following equation: [28,29,30,31]:

(2) $$\mathrm{Pure}\;\mathrm{water}\;\mathrm{flux}=\frac{V}{\mathrm{AtP}}$$

#### 2.5.2. Membrane Fouling Experiments 

The dynamic fouling tests for the polyethylene membranes were investigated using 15, 25, 35, and 45 ppm of the Humic Acid (HA) foulant. Three fouling cycles were conducted per experiment. Basically, the first cycle included the evaluation of pure water flux before fouling (J_1_) for pure distilled water for 1 h, followed by the second cycle that includes the foulant in the feed resulting in the foulant flux (J_p_). Finally, the third cycle was performed by backwashing the membrane using deionized water without applying any forms of external heat. The pure water flux after fouling (J_2_) was recorded. The following fouling ratios were calculated to assess the antifouling properties of the polyethylene membrane in the pilot DCMD system:

(3) $$\mathrm{Flux}\;\mathrm{recovery}\;\mathrm{ratio},\;\mathrm{FRR}\;[\%]=\left(\frac{{\;J}_{2}}{J_{1}}\right)\times 100$$

(4) $$\mathrm{Reversible}\;\mathrm{fouling}\;\mathrm{ratio},\;R_{r}\;[\%]=\left(\frac{J_{2}-{\;J}_{p}}{J_{1}}\right)\times 100$$

(5) $$\mathrm{Irreversible}\;\mathrm{fouling}\;\mathrm{ratio},\;{\mathrm{Rir}}_{\;}[\%]=\left(1-\frac{J_{2}}{J_{1}}\right)\times 100$$

(6) $$\mathrm{Total}\;\mathrm{fouling}\;\mathrm{ratio},\;R_{t}\;[\%]=\left(1-\frac{J_{p}}{J_{1}}\right)\times 100$$

#### 2.5.3. Energy Consumption Analysis

An important aspect of MD is the thermal energy consumption linked to the heat of the feed stream [9]. In this respect, the specific thermal energy consumption (STEC) is often used, calculated as the energy to supply to the feed recirculating inside the module, divided by the permeate production per time (kW h/m^3^) [4]:

(7) $$\mathrm{STEC}=\frac{Q_{f}\times {\;c}_{p}\times \left(T_{\mathrm{fin}}-T_{\mathrm{fout}}\right)}{Q_{p}}$$

where Q_f_ is the feed flow rate (kg/h), c_p_ is the specific heat of the feed (kJ/kg. K), T_fin_ is the feed temperature at the module inlet (K), T_fout_ is the feed temperature at the module outlet (K), and Q_p_ the permeate flow rate (L/h). Due to the fact that in DCMD processes, both the hot feed and the cold permeate water are brought into contact with the membrane under atmospheric pressure, the total pressure is assumed constant at 1 atm, resulting in a negligible viscous flow [32,33,34].

Another important performance indicator in an MD system is the specific electrical energy consumption (SEEC). It is defined as the amount of electrical energy consumed to produce a unit mass of pure water [7]. In the current MD pilot unit, the specific electrical energy consumption (SEEC) was calculated based on three main sources in the system: the permeate pump, the feed pump, and the unit’s control panel. When calculating the specific thermal and electrical energy consumptions, the specific energy consumption (SEC) (kWh/m^3^) is defined as the amount of total energy supplied (heat and electrical energy) to produce a unit mass of the product [35,36].

(8) SEC=STEC+SEEC

SEC was used in this work to evaluate the energy performance for a large pilot unit capacity of 207.31 m^3^/h.

### 2.6. Membrane Characterization

An optical contact angle meter (CAM) from DataPhysics Instruments GmbH (2013 model) was used to indicate the change in membrane pore wetting before, during, and after all DCMD tests. All measurements were made at a 1 μL dosing volume and 4 μL/s dosing rate for the liquid syringe injected with distilled water. Moreover, an FEI Quanta 200 environmental scanning electron microscope (SEM) and atomic force spectroscopy (AFM) were used to study the structural change on the membrane surface, providing deeper insights into the formation of crystals and accumulation of foulant on the membrane during all DCMD experimental work.

## 3. Results and Discussion

### 3.1. Long-Term MD Pilot Performance

#### 3.1.1. Membrane Surface Analysis

**Figure 3:**
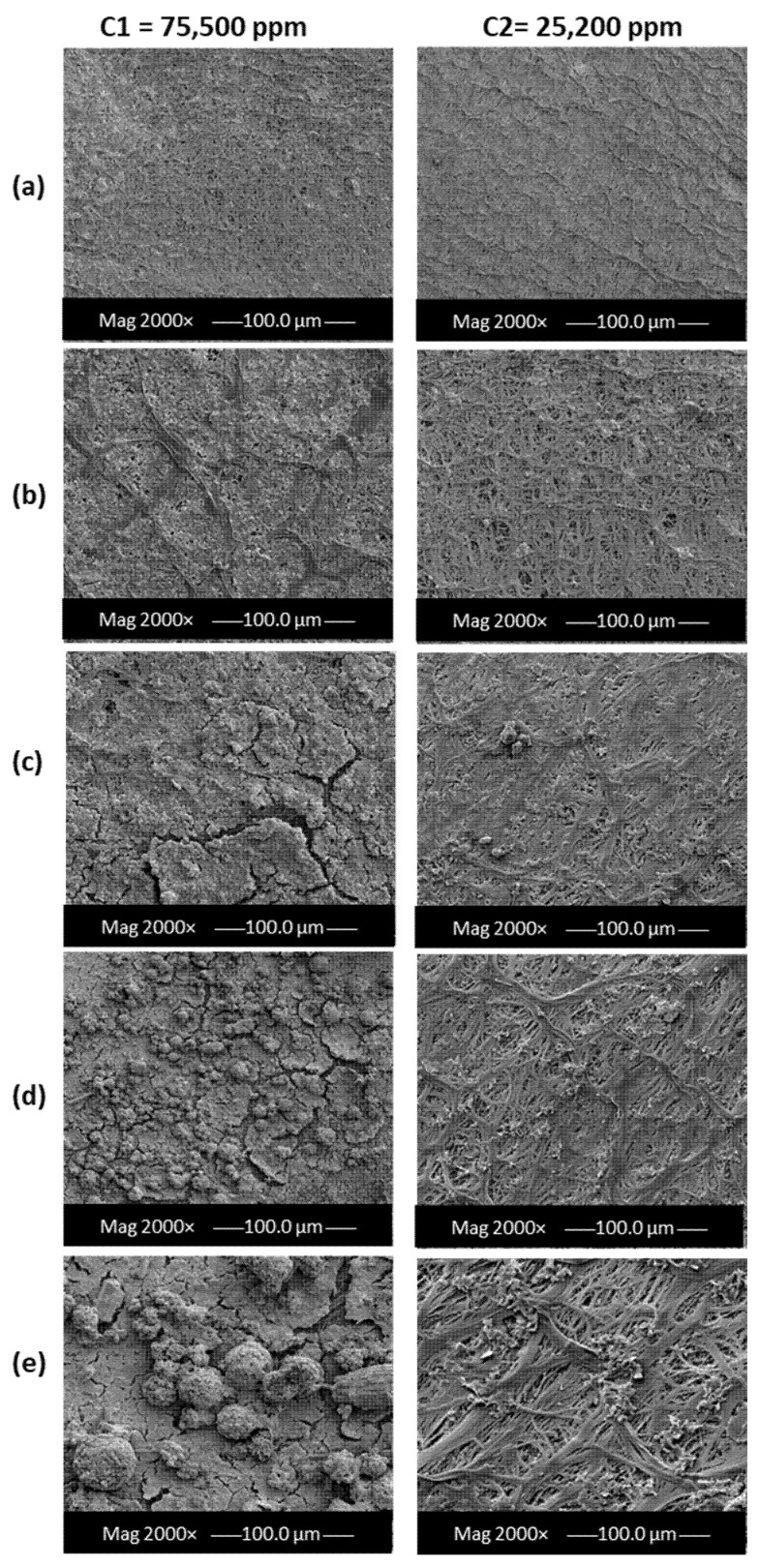
SEM images for PE membranes after (**a**) 20, (**b**) 40, (**c**) 60, (**d**) 80, and (**e**) 100 h of pilot-scale MD tests.

All membranes were dried immediately after removal from the DCMD pilot unit. Although pore wetting did not partially occur, the organic foulant has passed through the hydrophobic membrane leading to the proposal of an adsorption–desorption foulant migration mechanism. Despite the high hydrophobicity of polyethylene, there was a significant rise in the deposition of salt particles on the membrane’s feed side due to the immediate contact between the brine and membrane surface at harsh fouling operating parameters. SEM images for the membrane’s feed side were taken every 20 h during the entire duration of the experiment (Figure 3). 

**Figure 4:**
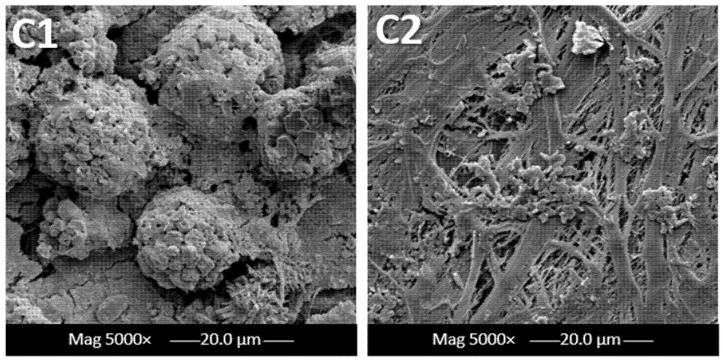
Magnified SEM images for PE membranes after 100 h at different concentrations immediately after pilot-scale MD tests.

It was confirmed that the amount of foulants penetrating through the membrane is largely dependent upon the adsorption strength of the membrane material. As a result, it hinders the membrane’s functionality in terms of the formation of cake layers and clogging of pores [37,38]. This can be clearly seen in the zoomed-in SEM images in Figure 4, where larger cake layers were formed due to the use of highly concentrated feed (C1) in comparison to less concentrated feed (C2). The formation of cakey layers and foulant depositions (also known as membrane scaling) can be avoided by physically eliminating the crystals deposited on the surface of the membrane, which often leads to the blockage of pores [14]. 

**Figure 5:**
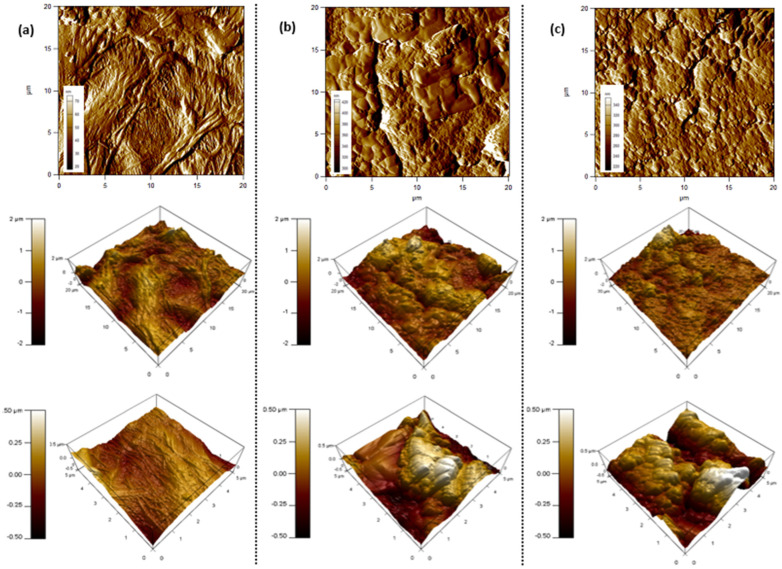
AFM images for PE membranes (**a**) before, (**b**) after 40 h, and (**c**) after 100 h of pilot-scale DCMD tests.

To further confirm the surface morphology with respect to increasing the duration of the pilot process, AFM inspection was conducted for the feed side of the membranes that were continuously exposed to the concentrated brine. The surface topography of the commercial polyethylene membranes was denoted in Figure 5, at scan rates of 1.0 Hz and scan sizes of 20 µm and 5 µm, respectively. The root-mean-square (RMS) roughness parameter was used as an indication of the material’s surface roughness [39]. The measured RMS values for all membranes prior to DCMD was 81.39 nm (Figure 5a). The surface roughness increased after 40 h of continuous testing, reaching a value of 213.1 nm (Figure 5b). A further increase in the RMS value was noted after 100 h of testing with a maximum roughness value of 357.1 nm (Figure 5c). The incremental increase is attributed to the accumulation of salt molecules and thereby the fouling of the membrane surface. Similar data trends were observed in previous studies [33,40]. 

**Figure 6:**
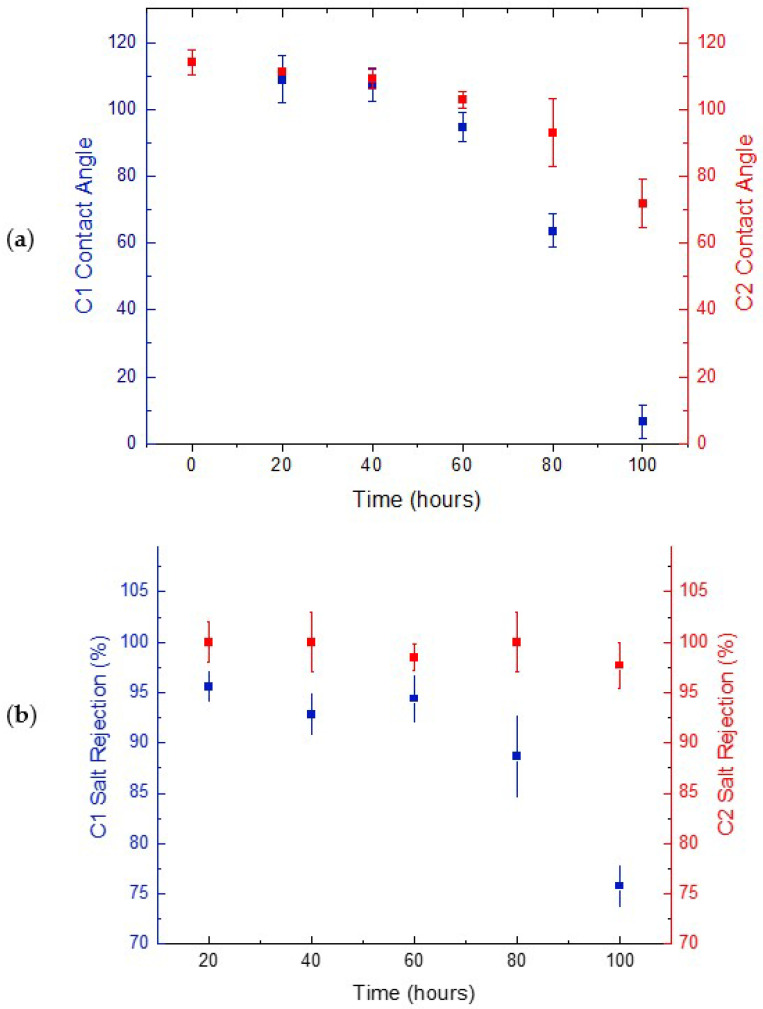
Contact angles (**a**) and salt rejections (**b**) of tested PE membranes during longtime DCMD operation at pilot scale.

**Figure 7:**
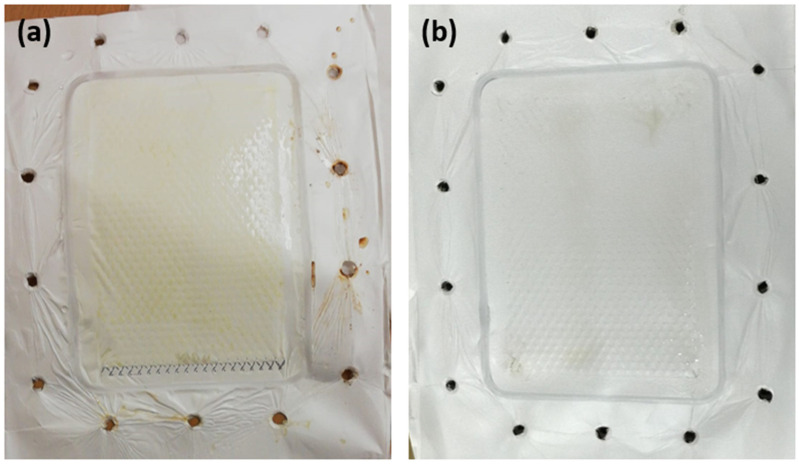
Images of PE membranes after 100 h of DCMD tests at concentrations of C1 and C2, (**a**) 75,500 and (**b**) 25,200 ppm, respectively.

During an MD operation, pore wetting is one significant issue that causes failure of the whole operation. During the desalination of synthetic brine, the hydrophobic tails of the existing amphiphilic molecules become interconnected with the hydrophobic membrane pore surface. This leaves the hydrophilic head exposed and thereby renders the hydrophilicity of the membrane pores. Hence, this instantly impacts the membrane wetting properties revealed in Figure 6a. At higher feed concentration, a much higher reduction was shown throughout the whole duration of MD pilot testing. A 4.3% loss in hydrophobicity was noted after the first 20 h, followed by a total of 94% reduction after the 100 h of testing was completed. In contrast, at a lower concentration, a lower drop of 2.6% was observed after the first 20 h, followed by a 37% decrease in the contact angle measurements. This huge reduction in the membrane’s wetting behaviour for C1 compared with C2 emphasizes that, due to long periods of immediate exposure to highly concentrated feed, its pores have already become saturated with chemical compounds and salts, causing an increase in adhesive forces between the water molecules and the polyethylene molecules on the membrane surface. These adhesive forces become greater than the cohesive forces within the water molecules. The existence of surfactants and organic compounds in the brine is a major contributor to pore wetting of the membranes was studied thoroughly in previous studies [41]. This significant absorption of brine into the membrane pores has thereby undermined the salt rejection rates denoted over a long period of time, as seen in Figure 6b. Similar to wettability analysis, the drop percentage in salt rejection rates showed a linearly proportional relation with feed concentration. The evaluation of salt rejection rates in membrane distillation studies is actually complicated and requires a systematic approach due to many determining factors. This includes the feed solution, membrane properties, and operating process conditions [42]. In this work, the only changing variable was the feed solution, while other factors remained fixed. The interplay between various factors that affect salt rejection in membrane distillation is dependent on whether the feed solution involves inorganic salts and organics. The presence of high amounts of salts and chemicals in the higher feed solution resulted in a higher potential for membrane scaling. Hence, a denser fouling layer at the membrane surface was produced, which therefore lowered the liquid surface tension and reduced the salt rejection compared to the moderate saline feed. The reduction in salt rejection rates for higher concentration feed was 21% in comparison with the lower ones of 2.3% during the entire MD operation process. This was visually reflected in Figure 7, showing the buildup of dense cake as confirmed by Vigneswaran and Kwon [43]. 

#### 3.1.2. Membrane Performance at Pilot-Scale

**Figure 8:**
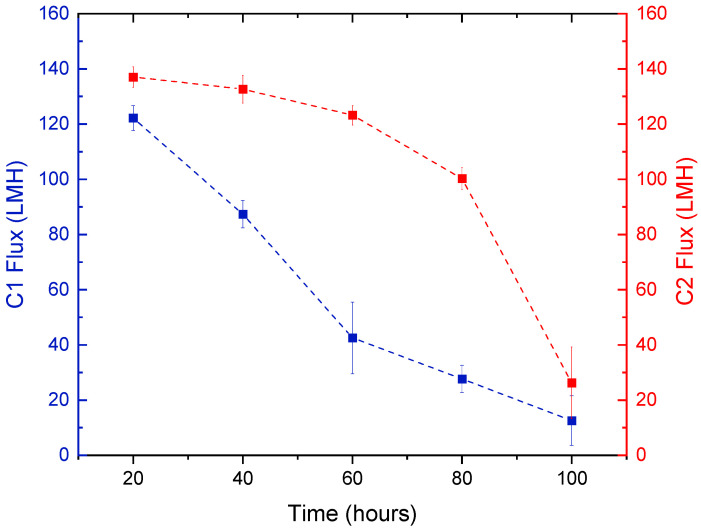
The effect of brine concentration on the permeate flux at longtime DCMD operations.

In all MD operations, successful membranes are the ones that show enhanced performance over a long period of time with high stability. In this work, the commercial PE membranes were tested at equal permeate and feed flow rates of 70 LPH with effective areas of 0.01 m^2^ and were noticeably affected by the 100-h experiment. The effect of varying concentrations on the water flux can be clearly seen in Figure 8, where both types of concentrated brines have eventually yielded a significant decline. The higher brine concentration of 75,500 ppm showed a rapid 20% flux reduction compared to the 25,200 ppm brine with only 3% reduction after the first 20 h. Similarly, after 100 h of intensive pilot-scale MD operation, the flux decline was nearly 90% and 80% for the highest and lowest feed concentrations, respectively. The decline in flux for C2 after 80 h of continuous MD operation indicates that, at given operating parameters, the membrane was no longer able to maintain its hydrophobicity with time. This caused a collapse of membrane pores leading to pore blockage. This comes into agreement with previous studies where the flux reduction was majorly due to intensified velocity on both sides of the membrane that consequently led to pore wetting [26]. Moreover, a decline in permeate flux may also be attributed to membrane fouling over long durations of MD tests [6]. Furthermore, the reduction in water vapor pressure caused by continuous exposure to highly concentrated feed led to the decline in the permeate flux.

Zuo et al. [44] tested the durability of PE membranes at long-term operating conditions using a 3.5 wt% NaCl feed solution at constant operating process parameters. A high flux (123.0 L/m^2^·h) was achieved at a high membrane thickness of 50 µm. McGaughey et al. [45] used commercial PTFE membranes using a bench-scale DCMD system designed for continuous long-term operation and showed that maintaining distillate-side hydrophobicity and/or internal hydrophobicity may be more important for long-term performance in an MD system. Another study by Mansour and Hasan [31] utilized real rejected brine using a pilot-scale DCMD unit. Each experiment lasted for 4 h. There was a 69% decline in the flux due to fouling formation with an optimum permeate flux and salt rejection of 16.7 LMH and 99.5%, respectively. 

### 3.2. Membrane Fouling Analysis

When a solution containing foulants is used as feed, a fouling layer is formed on the membrane surface by foulants deposition [46]. Crystals formed following MD applications lead to clogging of membrane pores and continue to grow within the pores, which allows penetration of foulants from the feed through the membrane leading to membrane wetting. Actually, the adsorption of foulants onto the surface or inside the pores of the membrane causes a rapid decline in the membrane flux, hindering the overall MD performance resulting in direct contamination of the permeate [14,40,47]. 

**Figure 9:**
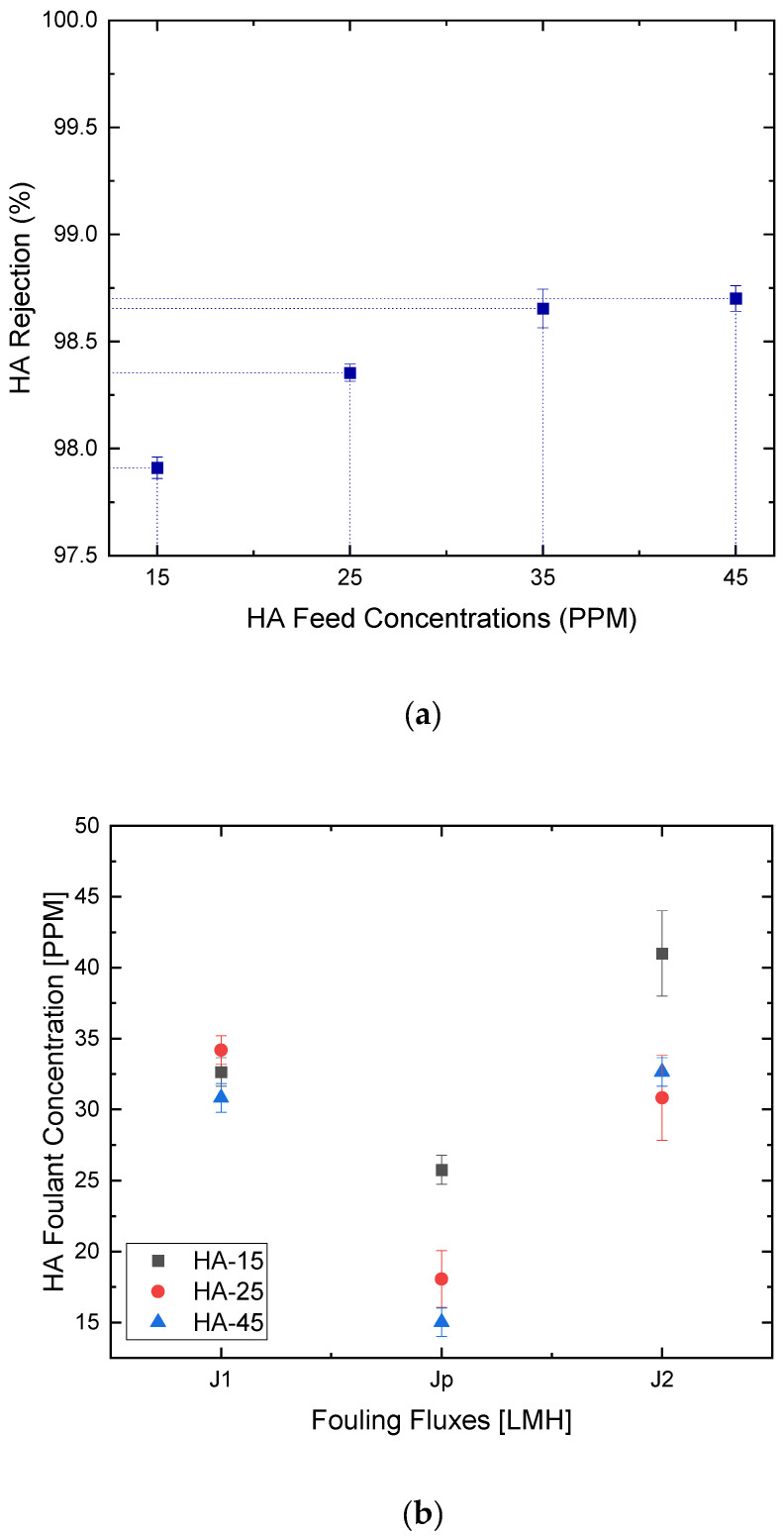
(**a**) HA rejection rates at different HA feed concentrations and (**b**) calculated fouling fluxes at optimum HA foulant concentrations.

As depicted in Figure 9a, at concentrations exceeding 25 ppm, the measured concentration reached 0.013 ppm with minimal change even at increasing foulant concentration up to 45 ppm. This indicates that a small change in the foulant’s concentration will result in a significant change in fouling behaviour on the membrane’s surface during the microfiltration process. In this work, the rejection rates of the HA foulant exceeded 98.5%. Similarly, Khayet et al. reported at least 96% of Humic Acid rejection using commercial PVDF membranes [48]. Previous studies have also reported similar findings [37]. As seen in Figure 9b, the fouling flux became much less than that of pure water flux. Such fouling behavior is largely dependent upon the consecutive absorption and deposition of HA particles onto the membrane surface [49]. 

**Figure 10:**
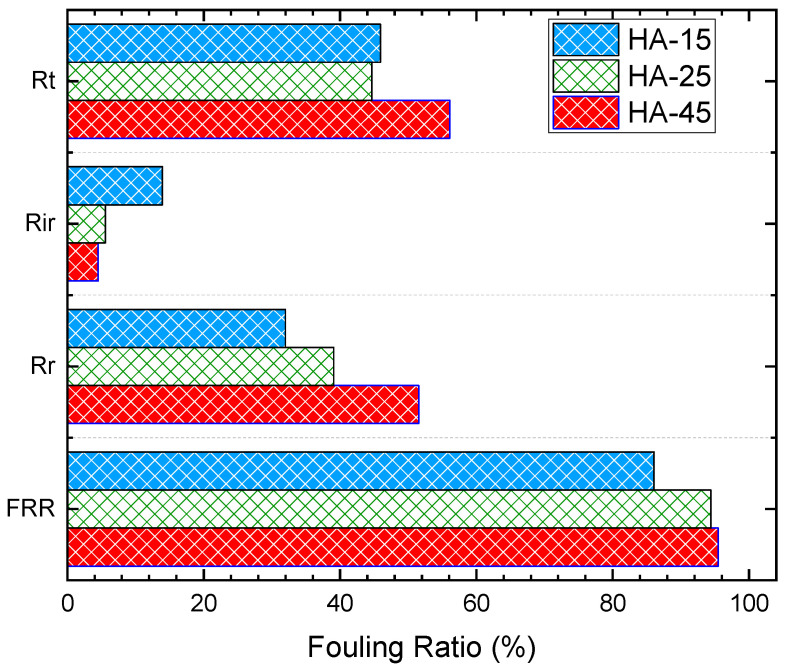
Calculated fouling ratios at increasing HA concentrations of 15, 25, and 45 ppm.

**Figure 11:**
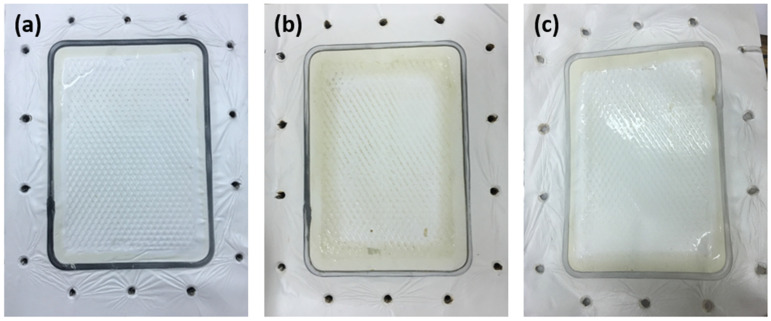
Visual effect of HA foulant on the membrane surface; (**a**) after the first wash with DI feed, (**b**) application of HA feed, (**c**) washing the foulant off the membrane using DI feed.

At increasing foulant concentrations, the foulant flux significantly declined by 21%, 47%, and 51%, respectively (Figure 9b). As confirmed by Cho et al., It was demonstrated that Humic Acid enhanced CaCO_3_ deposition on the membrane surfaces, thereby expediting the scaling phenomenon [50]. Similarly, previous studies have shown that HA fouling of membranes causes a rapid and irreversible loss of flux through the membrane, which limits the successful application in water or wastewater treatment technologies [51,52,53]. However, interestingly in this study, the irreversible fouling ratio was only 4.5% for the 45 ppm compared to 14% for the 15 ppm of HA feed. This implies that in our pilot MD system, hydraulic cleaning is not much needed for the elimination of HA adsorbed on the membrane. This also comes into agreement with the high flux recovery and reversible fouling ratios reaching up to 95% and 52%, respectively, at higher HA concentrations (Figure 10). This indicates that only water flushing would be sufficient for foulant removal in our MD pilot unit, as visually proven in Figure 11a–c. In fact, the increased R_r_ of membranes at increasing HA concentrations created a reversible adhesion between the polyethylene membrane surface and the HA molecules [51]. 

Srisurichan et al. [52] studied the fouling mechanism of hydrophobic PVDF membranes and used the cake filtration model to describe the HA aggregates on the membranes. The rise in total resistance was attributed to the existence of fouling layer resistance which significantly increased over time. Hence, a critical parameter to consider in terms of examining the anti-fouling performance against the membrane surface would be the adhesion forces at the interfaces [54,55]. As confirmed previously, with increasing feed concentrations, the electrostatic repulsive forces of HA with both HA and PE membranes promote the accumulation of the HA molecules resulting in a denser HA fouling layer on the membrane. 

### 3.3. Specific Energy Consumption (SEC) of DCMD Pilot Unit

**Figure 12:**
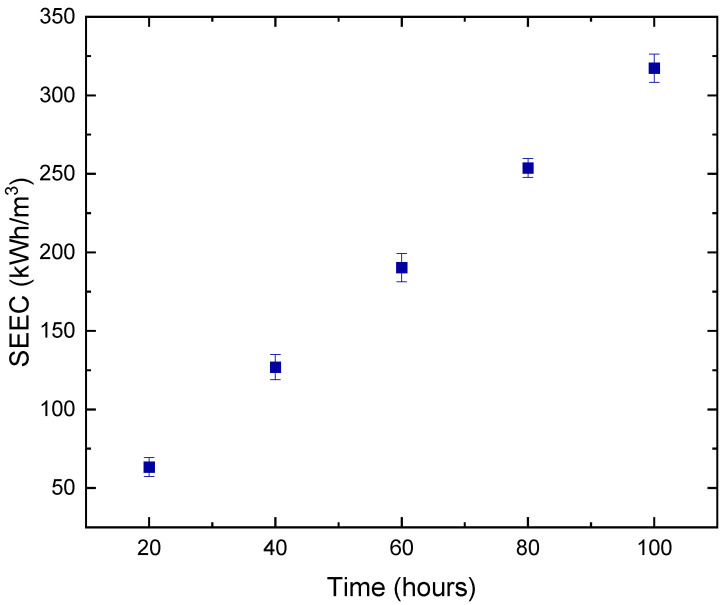
Specific electrical energy consumption calculated over time for MD pilot unit.

As a result of membrane fouling, there is a rapid increase in the optimum energy required for desalination either by decreasing productivity (flux) or increasing the required driving force [56]. Over the period of 100 h of continuous MD process, a positive linear relationship between both SEEC and the number of operating hours can be noted in Figure 12. This was an expected behavior as more electrical energy is required to provide more power for the entire duration of MD. Currently, the industry is focusing on using equipment with minimal usage of power at the pilot level [57]; however, the challenge still arises in the uncertainties of the energy usage within the specific boundaries of the entire MD pilot unit. Assuming continuing operation over a minimum of 20 h per day, the total electrical energy consumption for one pump, including pilot unit capacity, would be 131.01 kWh. Moreover, since the operation of MD at a large scale is performed based on two pumps for both the heating feed and cooling permeate tanks, then the total specific electrical energy consumed for the whole pilot unit would be 138.61 kWh/m^3^. 

**Figure 13:**
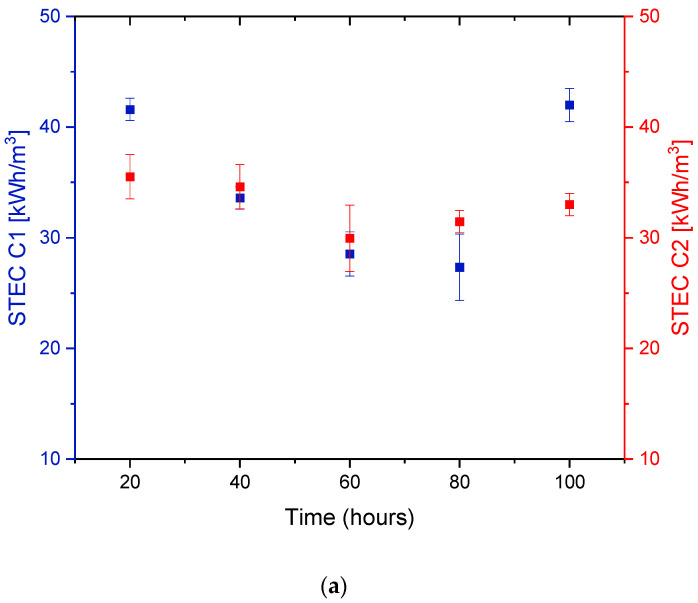

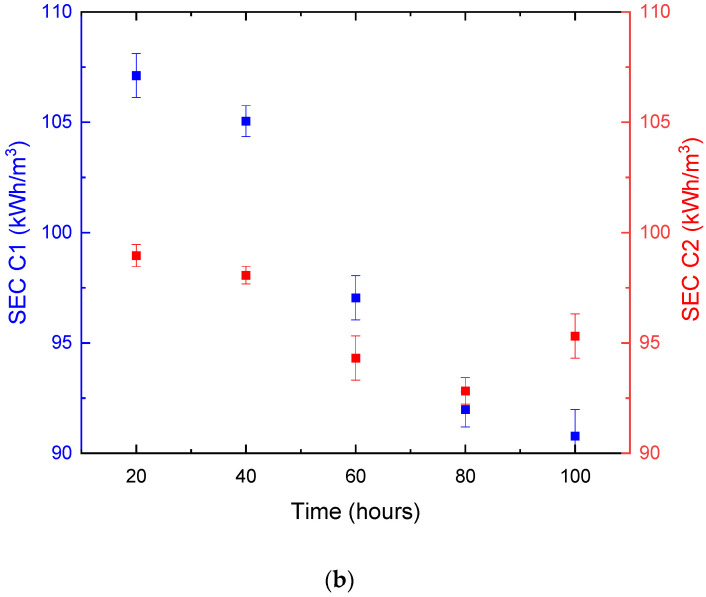
Influence of different feed concentrations on (**a**) specific thermal energy consumption and (**b**) specific energy consumption for the entire MD operation process in pilot scale.

Generally, in DCMD, the pumps, electrical heating inside the feed tank, and external water coolant running through the coolant spiral inside the distillate tank, all lead to the consumption of electrical energy. In Figure 13, the overall heat input raised, resulting in an increase in the pilot system’s STEC from starting points of 35.51 and 43.667 kWh/m^3^, for C2 and C1, respectively. This agrees with some findings in the literature [58]. 

Feed salinity plays a significant role in determining the thermal energy consumption of an MD system. With the reduction in feed concentration, as presented in Figure 13a, there was a 19% drop in STEC after the first 20 h. The total reduction in STEC at higher feed concentrations was greater than that of lower concentrations, with 37% and 21%, respectively. This was due to the direct effect of feed salinity on the viscosity and mobility of the stream passing through the membrane, which in turn affects the vapor pressure of the solution [59]. In this work, the feed and permeate inlet temperatures were fixed at T_Fin_ = 70 °C, T_Pin_ = 20 °C, respectively. The flowrates of Q_F_ = Q_P_ = 70 LPH were equal for both the feed and permeate streams, respectively. The specific heat capacity was assumed constant in the whole pilot system. The minimal values of the STEC between 40 and 90 h of continuous DCMD pilot operation were reached as a result of the fouling effect on the system’s thermal performance. The formation of the fouling layers on the membrane surface raised the overall membrane thermal resistance and lowered its overall heat transfer coefficient. As depicted earlier in Figure 3 and Figure 4, as a function of operating time, an increase in the attachment of foulants onto the membrane surface took place. In the presence of inorganic compounds and chemical substances, both high ionic strength and large particles concentrations increased the membrane fouling to an extent where water flux rapidly declined. At fixed inlet conditions, the increased buildup of the fouling layers acted as extra resistance to heat transfer and hindered the effectuality of the pilot system. This, in turn, intensified the amount of consumed energy in the pilot system; hence the STEC value increased. Interestingly, the total SEC reduction after 100 h of pilot operation at lower feed concentration was only 3% compared to that of 15% at higher concentration (Figure 13b). The depicted outcomes provided a better insight into the relationship between membrane fouling and specific energy consumption.

Previous studies have worked on the reduction in energy consumption and thus improving the thermal efficiency of the MD operation by linking the DCMD with a whole heat exchanger for latent heat recovery [60] and by brine recycling for water recovery purposes [61], where optimization between feed and distillate flow rates is necessary for lower energy consumption.

Table 2 lists the findings of this work and compares them with previous attempts on DCMD systems using different types of membranes at different operating parameters. Despite the large unit capacity of our system, we were still able to achieve an optimum specific energy consumption of 107.1 kWh/m^3^ and 90.8 kWh/m^3^ after 20 h and 100 h of membrane exposure to different feed concentrations, respectively. This signifies an optimum energetic performance for the DCMD pilot unit used in this study. However, the lack of clarity on whether only the energy used by the main equipment should be included or excluded in the analyses is one form of arising uncertainties, especially at the process level [62]. Thus, comparing the results of this work effectively with other studies is challenging [7]. 

SEC is generally used for benchmarking energy use in industrial processes to compare and evaluate the overall MD energy performance. The age of equipment and the capacity of the pilot unit are a few factors that influence the SEC. For instance, while newer equipment is more likely to be more energy-efficient compared to older equipment, it may require several years for optimization of newly installed equipment. This work showed optimum SEC achieved with higher permeabilities at much lower MD duration in comparison with other systems operating at higher times and at lower unit capacities. This variation can be attributed to many factors, such as the location, size, and rate of permeate production used for an MD pilot system [66,67,68].

The considerably high amounts of energy consumption (ranging from 95.3 kWh/m^3^ to 107.1 kWh/m^3^) that are required to produce permeate with drinking water standards signify the importance of utilizing low-grade thermal energy by creating a hybrid MD-integrated system that outperforms other desalination systems. Results obtained from this work can create a solid basis to identify ways of enhancement in the energy efficiency for full-scale industrial processes. 

Furthermore, one of the main statistical measuring elements was used in this study to better investigate the strength of the linear relationships of all data sets. The Pearson Correlation was used as per the following equation [30]:

(9) $$\mathrm{Correl}\left(A,B\right)=\frac{\mathrm{Covariance}\left(A,B\right)}{\mathrm{Std}.\mathrm{Dev}\;A\;\times \mathrm{Std}.\mathrm{Dev}\;B}$$

**Figure 14:**
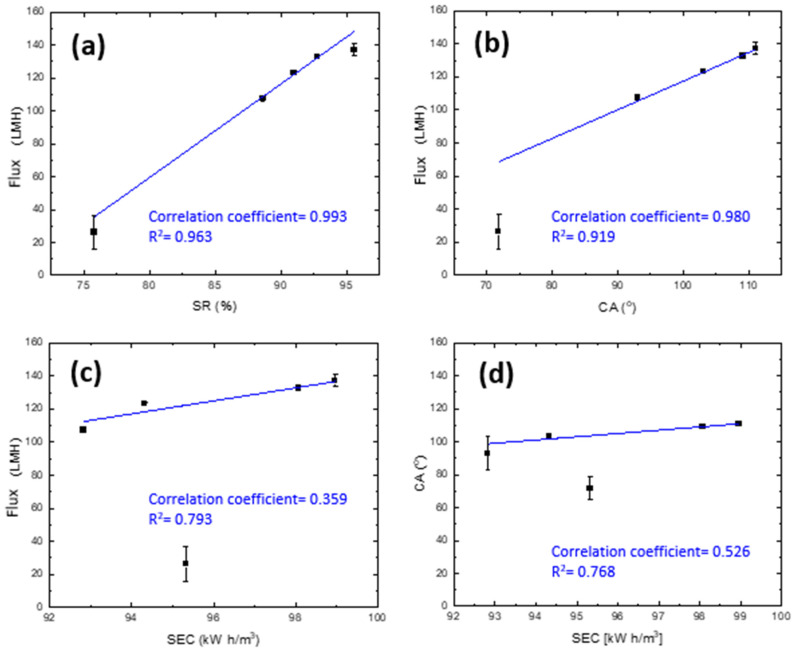
The correlations of the calculated water flux with (**a**) salt rejection, (**b**) contact angle, (**c**) specific energy consumption, and (**d**) the correlation of the contact angle with the specific energy consumption.

In this study, the outcome of all correlations is plotted in Figure 14. The correlation results suggest that the permeate flux is greatly affected by the percentage of salt rejection (Figure 14a) as well as the water contact angle of the membrane (Figure 14b), with coefficients of 0.963 and 0.919, respectively. Furthermore, a moderate positive correlation was found for the plot relating CA with SEC (Figure 14d). However, a weak correlation factor of 0.359 was depicted for the flux with respect to STEC (Figure 14c). This may prove that there is a nonlinear relationship between both parameters but does not necessarily imply that there is no relationship between both sets of data. In fact, despite the low correlation, a directly proportional relation can still be seen at 40 h and beyond the whole duration of the DCMD process. Generally, the outcomes of this work have not shown any negative correlation coefficients, which proves that all sets of data obtained from this study are moving in the same linear direction. The coefficient of determination (R^2^) was also calculated, showing a data-relation magnitude ranging from 0.768 to 0.993. This showed a high variance proportion in the dependent variables predicted from the independent variables [69]. 

## 4. Conclusions

The selection of a proper hydrophobic membrane is essential to guarantee the development of technologies, especially those used in water treatments for the oil and gas industries. The long-term performance of a pilot DCMD system was investigated in this study for polyethylene membranes and has shown high energetic performance and different saline levels of the feed. High flux recovery ratios of 86%, 94%, and 95% were depicted at increasing HA concentrations of 15, 25, and 45 ppm, respectively. However, after 40 h of direct membrane exposure with saline feed, HA foulant particles on the membrane surface started to form cake layers causing a decline in the permeate flux. Towards the end of the entire MD operation of 100 h, the fouling pattern became evenly distributed within the grains of the membrane, reducing its pore size and therefore leading to pore wetting. The findings from this work come in line with recent technological innovations and are largely accountable for the optimization of industrial MD processes during the treatment of industrial wastewater.

## Figures and Tables

**Table 1:** Detailed chemical constituents in prepared thermal brine [26].

Chemicals	Feed Composition [g/L]	
	C1	C2
Na	23,876	11,938
Mg	2520	1260
Ca	765	382
K	793	396
Sr	11	5
B	9	4
Cl	42,682	21,341
SO_4_	4229	2114
HCO_3_	726	363
Br	67	33

**Table 2:** PWP and SEC values recorded in previous studies for DCMD systems only.

Membrane	Feed Type	Temperature Inlets [°C]		Duration of MD [hours]	PWP [LMH]	SEC [kWh/m^3^]	Plant Capacity [m^3^/h]	Ref
		Feed	Permeate					
PP	Distilled Water	59.2	14.3	3	56.2	3550–4580	-	[63]
PVDF	Simulated RO Brine	80	30	-	10.80–12.6	130–1700	-	[64]
PTFE	Wastewater	60	18–21	840–1800	2–5	1500	3.85	[65]
PE	Synthetic Brine	70	20	20	122.2	107.1	207.31	This work
				100	12.6	90.8		

## Data Availability

The datasets generated and analyzed during the current study are available from the corresponding author on reasonable request.
